# Molecular Mechanisms of Carbapenem Resistance in Clinical Enterobacterales Isolates: The Role of Carbapenemases and Porin Loss

**DOI:** 10.7759/cureus.110858

**Published:** 2026-06-14

**Authors:** Pavithraa Suresh, Muthamizhveena R, Guruswamy Narayanan Prabhu, Manchireddy Manish, Jigar Gusani, Dipin Kumar P U, Sajidali S Saiyad

**Affiliations:** 1 Department of Microbiology, Sri Venkateshwaraa Medical College Hospital and Research Centre, Puducherry, IND; 2 Department of Pharmacology, Government Medical College, Pudukkottai, Pudukkottai, IND; 3 Department of Cardiothoracic Surgery, Sir Ivan Stedeford Hospital, Chennai, IND; 4 Department of Anesthesiology, Madha Medical College and Research Institute, Chennai, IND; 5 Department of Microbiology, Dharmsinh Desai University, Dr N. D. Desai Faculty of Medical Science and Research, Nadiad, IND; 6 Department of Internal Medicine and Infectious Diseases, Malabar Institute of Medical Sciences (MIMS) Hospital, Kozhikode, IND; 7 Department of Physiology, Pacific Medical College and Hospital, Pacific Medical University, Udaipur, IND

**Keywords:** antimicrobial resistance, bla_kpc, bla_ndm, carbapenemase genes, carbapenem resistance mechanisms, carbapenem-resistant enterobacterales, molecular epidemiology, outer membrane porins

## Abstract

Background and objective

Carbapenem-resistant Enterobacterales (CRE) have emerged as a major global public health threat, driven by the rapid dissemination of carbapenemase genes that compromise the efficacy of last-line antimicrobial agents. Comprehensive characterization of these mechanisms is essential for informing antimicrobial stewardship and infection-control strategies. The objective of this study was to investigate the molecular mechanisms underlying carbapenem resistance among clinical Enterobacterales isolates, with particular emphasis on carbapenemase genes and outer membrane porin expression alterations.

Methods

A retrospective cross-sectional study was conducted on 120 non-duplicate CRE isolates recovered from clinical specimens at a tertiary care hospital in Rajasthan, India, between January 2024 and December 2025. Species identification was performed using MALDI-TOF mass spectrometry, while antimicrobial susceptibility testing and carbapenem minimum inhibitory concentration (MIC) determination were carried out according to CLSI guidelines. Carbapenemase production was assessed using the Carba NP test. PCR was used to detect major carbapenemase and extended-spectrum β-lactamase (ESBL) genes. Outer membrane porin expression was evaluated by SDS-PAGE with densitometric analysis.

Results

*Klebsiella pneumoniae* accounted for the majority of isolates (75.0%), and most originated from intensive care units (54.2%). Carbapenemase activity was detected in 85.0% of isolates, while 87.5% harbored at least one carbapenemase gene. The most prevalent genes were bla_NDM (50.0%), followed by bla_KPC (16.7%) and bla_OXA-48-like (16.7%). ESBL genes were highly prevalent, particularly bla_CTX-M (79.2%). A marked reduction in OmpK36 expression was observed in 62.5% of isolates, and concurrent OmpK35/OmpK36 reduction occurred in 25.0%. Isolates with both carbapenemase genes and porin expression reduction demonstrated higher imipenem and meropenem MICs compared with carbapenemase-positive isolates without porin reduction (Mann-Whitney U test: for imipenem, U = 1420, Z = -5.02, p < 0.001; for meropenem, U = 1385, Z = -5.18, p < 0.001).

Conclusions

Carbapenem resistance in clinical Enterobacterales is strongly associated with carbapenemase production, particularly bla_NDM and bla_KPC, and is augmented by reduced outer membrane porin expression. These findings highlight the importance of integrating molecular diagnostics with routine susceptibility testing to guide antimicrobial stewardship and infection-control interventions.

## Introduction

Antimicrobial resistance (AMR) is a major global health challenge associated with increased morbidity, mortality, and healthcare expenditure due to limited treatment options [1]. Among these organisms, carbapenem-resistant Enterobacterales (CRE) represent a particularly important threat because of their ability to cause severe healthcare-associated and community-acquired infections and to disseminate resistance determinants rapidly through mobile genetic elements [2].

Carbapenems are key drugs for multidrug-resistant Gram-negative infections, but rising resistance has reduced their clinical effectiveness worldwide [3,4]. Among the various mechanisms contributing to carbapenem resistance in Enterobacterales, carbapenemase production remains the most clinically relevant, particularly KPC, NDM, VIM, and OXA-48-like enzymes [5]. These resistance genes are frequently carried on plasmids, transposons, and other mobile genetic elements, facilitating rapid horizontal transmission across bacterial species and healthcare environments [6].

In addition to carbapenemase production, several non-carbapenemase mechanisms contribute to resistance. Reduced permeability caused by reduced expression or alteration of outer membrane porins, particularly OmpK35 and OmpK36 in *Klebsiella pneumoniae*, decreases antibiotic influx and may substantially increase carbapenem minimum inhibitory concentrations (MICs), especially when combined with extended-spectrum β-lactamase (ESBL) or AmpC β-lactamase production [7]. Furthermore, overexpression of multidrug efflux systems such as AcrAB-TolC may contribute to reduced intracellular antibiotic accumulation and enhanced resistance phenotypes [8]. Increasing evidence suggests that the interaction between carbapenemase production and porin alterations plays a critical role in determining the magnitude of carbapenem resistance and clinical treatment failure [9].

Recent studies have demonstrated substantial geographical variation in the molecular epidemiology of CRE. While KPC-producing strains predominate in several regions of North America and Europe, NDM-producing Enterobacterales are increasingly reported across Asia, particularly in India and neighboring countries [10,11]. Indian Council of Medical Research surveillance reports substantial carbapenem resistance in India, affecting about 30% of *Escherichia coli *and 58% of *K. pneumoniae i*solates [12]. A recent systematic review and meta-analysis from India further demonstrated significant regional variation in carbapenem resistance prevalence and resistance gene distribution, emphasizing the need for institution-specific surveillance and molecular characterization studies [13].

Despite growing recognition of CRE as a critical healthcare threat, most available studies from India primarily focus on phenotypic resistance patterns or the prevalence of individual carbapenemase genes. Comprehensive investigations integrating phenotypic carbapenemase detection, molecular characterization of resistance determinants, and analysis of outer membrane porin expression remain limited, particularly at the institutional level. Consequently, the relative contribution and interaction of these resistance mechanisms within specific clinical settings remain incompletely understood.

We hypothesized that carbapenem resistance among clinical Enterobacterales isolates is predominantly mediated by carbapenemase production and is further enhanced by concomitant outer membrane porin expression alterations. Therefore, the present study aimed to characterize the molecular mechanisms of carbapenem resistance among non-duplicate clinical Enterobacterales isolates by identifying major carbapenemase genes, evaluating outer membrane porin expression, and assessing their association with carbapenem resistance levels.

## Materials and methods

This retrospective cross-sectional observational study was conducted in the Department of Microbiology of a tertiary care hospital in Rajasthan, India, between January 2024 and December 2025. The study was designed to investigate the molecular mechanisms responsible for carbapenem resistance among clinical Enterobacterales isolates recovered during routine diagnostic microbiology services. Institutional Ethics Committee approval was obtained prior to commencement of the study (approval no. PMU/PMCH/IEC/GEN/2026/295; dated March 31, 2026). Because only anonymized laboratory isolates and routinely collected microbiological data were analyzed, the Ethics Committee waived the need for individual informed consent. A total of 120 non-duplicate CRE isolates were included in the study. Consecutive sampling was employed, and all eligible isolates recovered during the predefined study period were analyzed. As all eligible isolates were analyzed, a formal sample size calculation was not performed for this retrospective descriptive study.

Clinical isolates were recovered from routine patient specimens, including blood, urine, respiratory secretions, pus, wound swabs, and other clinically significant samples submitted to the microbiology laboratory. We analyzed non-duplicate *K. pneumoniae*, *E. coli*, and *Enterobacter cloacae *complex isolates resistant to at least one carbapenem (imipenem, meropenem, or ertapenem) per Clinical and Laboratory Standards Institute (CLSI) 2022 standards. Surveillance, environmental, and incomplete records were excluded.

Duplicate isolates from the same patient, surveillance cultures, environmental isolates, screening specimens, and isolates associated with incomplete microbiological or demographic records were excluded from the study. Isolates were identified by MALDI-TOF mass spectrometry (VITEK^®^ MS, bioMérieux, Marcy-l’Étoile, France). Antimicrobial susceptibility testing was performed using the VITEK^®^ 2 Compact system, and MICs for imipenem, meropenem, and ertapenem were confirmed by broth microdilution following CLSI 2022 recommendations. Quality control employed *E. coli *ATCC 25922 and *Pseudomonas aeruginosa *ATCC 27853 reference strains.

Carbapenemase activity was evaluated using the Rapidec^® ^Carba NP assay (bioMérieux) according to manufacturer and CLSI recommendations. Genomic DNA was extracted from overnight cultures, and PCR assays targeting bla_KPC, bla_NDM, bla_VIM, bla_IMP, bla_OXA-48-like, bla_CTX-M, bla_SHV, and bla_TEM were performed using previously validated laboratory protocols routinely employed for carbapenemase and ESBL gene detection. Appropriate positive and negative controls were included in each run. Internal laboratory quality-control procedures were followed throughout testing, including verification of control performance and review of amplification results before interpretation. Amplified products were analyzed by electrophoresis on 1.5% agarose gels and visualized under ultraviolet illumination. PCR products were identified based on expected amplicon sizes. Sequencing confirmation of amplified products was not performed because the study relied on archived retrospective laboratory data.

Outer membrane porin analysis

Outer membrane proteins (OMPs) were extracted using the sodium lauryl sarcosinate extraction method as previously described. Protein concentrations were standardized to 20 µg per lane prior to electrophoretic analysis to minimize variability between samples. OMPs were separated by sodium dodecyl sulfate-polyacrylamide gel electrophoresis (SDS-PAGE) on 12% polyacrylamide gels and stained with Coomassie Brilliant Blue R-250. Porin expression was evaluated by comparison with reference strains exhibiting normal porin expression and with reported molecular weight ranges for Enterobacterales porins. In *K. pneumoniae* isolates, bands corresponding to the expected molecular weights of OmpK35 and OmpK36 were assessed. For non-*Klebsiella* isolates, analysis was limited to major outer membrane porin bands of comparable molecular weight ranges, and specific porin identities were not confirmed.

Densitometric analysis was performed using ImageJ (NIH, Bethesda, MD, USA). Band intensities were normalized to the reference strain, and porin reduction was defined as expression below 30% of the control. Absence of a detectable signal was recorded as porin loss. All experiments were performed in duplicate.

Data management and statistical analysis

Data were analyzed using IBM SPSS Statistics for Windows, version 25.0 (released 2017; IBM Corp., Armonk, NY, USA). Categorical variables are presented as frequencies and percentages, while continuous variables are reported as mean ± SD or median (IQR), as appropriate. Normality was assessed using the Shapiro-Wilk test. Continuous variables were compared using the Mann-Whitney U test, where appropriate. Associations between resistance mechanisms and carbapenem MICs were evaluated using univariate analyses. Multivariable analyses were not performed because the study was designed as a descriptive retrospective investigation, and several resistance subgroups contained limited sample sizes.

## Results

Characteristics of the study isolates

Among 120 CRE isolates, *K. pneumoniae *accounted for 90 (75.0%), followed by *E. coli *(18 (15.0%)) and *E. cloacae *complex (12 (10.0%)). The majority originated from intensive care units (65 (54.2%)). General medical and surgical wards accounted for 40 isolates (33.3%), while outpatient departments contributed 15 isolates (12.5%). The detailed species and source distributions are presented in Table 1.

**Table 1:** Distribution of CRE isolates according to species and clinical source (N = 120) Data are presented as numbers (n) and percentages (%). N = total number of isolates analyzed. Statistical analysis: Descriptive statistics only; no inferential statistical comparisons were performed for species or source distribution. CRE, carbapenem-resistant Enterobacterales

Variable	Category	n (%)
Species	*Klebsiella pneumoniae*	90 (75.0)
	*Escherichia coli*	18 (15.0)
	*Enterobacter cloacae* complex	12 (10.0)
Clinical source	ICU	65 (54.2)
	General medical and surgical wards	40 (33.3)
	OPD	15 (12.5)

Phenotypic and molecular detection of carbapenem resistance mechanisms

Molecular analysis demonstrated the presence of at least one carbapenemase gene in 105 isolates (87.5%). Phenotypic screening using the Carba NP assay identified carbapenemase activity in 102 of 120 isolates, corresponding to a positivity rate of 85.0%. Among the 105 PCR-positive isolates, 102 were also positive by the Carba NP assay, whereas three isolates carrying carbapenemase genes were negative by phenotypic testing. No isolates were positive by the Carba NP assay in the absence of a detectable carbapenemase gene. Among the detected carbapenemase genes, bla_NDM was the most prevalent, identified in 60 isolates (50.0%). The bla_KPC gene was detected in 20 isolates (16.7%), while bla_OXA-48-like and bla_VIM were identified in 20 (16.7%) and 5 (4.2%) isolates, respectively. The coexistence of multiple carbapenemase genes was observed in 10 isolates (8.3%), with the most frequent combination involving bla_KPC and bla_NDM. Overall, carbapenemase-associated resistance determinants were identified in the majority of the isolates examined. The distribution of carbapenemase and ESBL genes is shown in Table 2.

**Table 2:** Distribution of carbapenemase and ESBL genes among CRE isolates (N = 120) Data are presented as numbers (n) and percentages (%). Percentages are calculated out of the total 120 isolates. Statistical analysis: Descriptive only; no inferential comparisons were performed. CRE, carbapenem-resistant Enterobacterales; ESBL, extended-spectrum β-lactamase; KPC, *Klebsiella pneumoniae *carbapenemase; NDM, New Delhi metallo-β-lactamase; OXA, oxacillinase; VIM, Verona integron-encoded metallo-β-lactamase

Gene category	Resistance gene	n (%)
Carbapenemase genes	bla_NDM	60 (50.0)
	bla_KPC	20 (16.7)
	bla_OXA-48-like	20 (16.7)
	bla_VIM	5 (4.2)
	Any carbapenemase gene	105 (87.5)
	Isolates with multiple carbapenemase genes (≥2)	10 (8.3)
ESBL genes	bla_CTX-M	95 (79.2)
	bla_TEM	70 (58.3)
	bla_SHV	55 (45.8)

In addition to carbapenemase genes, ESBL-associated determinants were frequently identified. The bla_CTX-M gene was the most common ESBL marker, detected in 95 isolates (79.2%), followed by bla_TEM in 70 isolates (58.3%) and bla_SHV in 55 isolates (45.8%).

Outer membrane porin expression alterations

Analysis of OMPs revealed a high frequency of porin expression alterations among carbapenem-resistant isolates. Reduction of OmpK36-associated porin bands, or corresponding major outer membrane porin bands in non-*Klebsiella *isolates, was observed in 75 (62.5%) isolates. Concurrent reduction of both OmpK35 and OmpK36 was identified in 30 isolates (25.0%). Porin expression alterations were detected in a substantial proportion of isolates carrying carbapenemase genes and were also present among isolates lacking detectable carbapenemase determinants.

Association between resistance mechanisms and carbapenem MICs

Carbapenemase genes were identified in 105 of 120 isolates (87.5%). Among carbapenemase-positive isolates, 69 (65.7%) exhibited porin reduction, whereas all carbapenemase-negative isolates demonstrated porin reduction. Carbapenemase-positive isolates with porin reduction showed substantially higher imipenem and meropenem MICs than carbapenemase-positive isolates without porin reduction. The distribution of resistance mechanisms, porin alterations, and carbapenem MIC values is summarized in Table 3. The association between porin reduction and carbapenem MICs among carbapenemase-positive isolates is presented in Table 4.

**Table 3:** Distribution of resistance mechanisms, porin reduction, and carbapenem mics among Enterobacterales isolates (N = 120) Data are presented as median with IQR and range for MIC values and as numbers (n) with percentages (%) for categorical variables. Porin reduction was defined as band intensity <30% of the normal control by densitometry. ^*^ 65.7% of carbapenemase-positive isolates (69/105) exhibited porin reduction. ^†^ 100% of isolates in this subgroup exhibited porin reduction by definition. ^‡^ 100% of carbapenemase-negative isolates exhibited porin reduction. Statistical analysis: Descriptive statistics only; no inferential statistical comparisons were performed for the distributions shown in this table. MIC, minimum inhibitory concentration

Resistance mechanism	n (% of total, N = 120)	Imipenem MIC (µg/mL), median (IQR) (range)	Meropenem MIC (µg/mL), median (IQR) (range)	Isolates with porin reduction, n (%)
Carbapenemase-positive (all)	105 (87.5)	12.0 (6.0-18.0) (4.0-64.0)	16.0 (8.0-24.0) (4.0-128.0)	69 (65.7)^*^
Carbapenemase-positive with porin reduction	69 (57.5)	18.0 (12.0-24.0) (8.0-64.0)	24.0 (16.0-32.0) (8.0-128.0)	69 (100.0)^†^
Carbapenemase-positive without porin reduction	36 (30.0)	4.0 (2.0-6.0) (2.0-8.0)	6.0 (4.0-8.0) (2.0-16.0)	0 (0.0)
Carbapenemase-negative	15 (12.5)	4.0 (2.0-6.0) (2.0-8.0)	6.0 (4.0-12.0) (2.0-16.0)	15 (100.0)^‡^

**Table 4:** Association between porin reduction and carbapenem MICs among carbapenemase-positive isolates Data are presented as medians with IQRs. Statistical analysis: Comparisons of MIC values between carbapenemase-positive isolates with porin reduction (n = 69) and carbapenemase-positive isolates without porin reduction (n = 36) were performed using the Mann-Whitney U test because MIC data were non-normally distributed. Isolates with porin reduction demonstrated significantly higher imipenem and meropenem MICs compared with isolates without porin reduction (p < 0.001 for both comparisons). Significance threshold: p < 0.05 was considered statistically significant. MIC, minimum inhibitory concentration

Resistance mechanism	n (%)	Imipenem MIC median (IQR), µg/mL	U-statistic	Z-score	p-value	Meropenem MIC median (IQR), µg/mL	U-statistic	Z-score	p-value
Carbapenemase-positive with porin reduction	69 (57.5)	18.0 (12.0-24.0)	1420	-5.02	<0.001	24.0 (16.0-32.0)	1385	-5.18	<0.001
Carbapenemase-positive without porin reduction	36 (30.0)	4.0 (2.0-6.0)	-	-	-	6.0 (4.0-8.0)	-	-	-

Carbapenemase-positive isolates with porin reduction demonstrated significantly higher imipenem MICs (median 18.0 µg/mL, IQR 12.0-24.0) compared with those without porin reduction (median 4.0 µg/mL, IQR 2.0-6.0). Similarly, meropenem MICs were higher in the porin reduction group (median 24.0 µg/mL, IQR 16.0-32.0) than in the group without porin reduction (median 6.0 µg/mL, IQR 4.0-8.0). Both differences were statistically significant (Mann-Whitney U test: for imipenem, U = 1420, Z = -5.02, p < 0.001; for meropenem, U = 1385, Z = -5.18, p < 0.001).

Summary of key findings

Carbapenemase genes were detected in 87.5% of isolates, with bla_NDM and bla_KPC representing the predominant resistance determinants. ESBL genes were highly prevalent, particularly bla_CTX-M. Porin expression alterations were observed in a substantial proportion of isolates, with OmpK36 reduction representing the most frequent abnormality. Isolates possessing both carbapenemase genes and porin expression reduction exhibited significantly higher carbapenem MIC values than carbapenemase-positive isolates without porin reduction (p < 0.001), as shown in Table 4.

## Discussion

CRE represent a major AMR threat because of their rapid dissemination, limited treatment options, and association with adverse clinical outcomes [1,2]. In this study, carbapenem resistance was predominantly driven by carbapenemase production, with bla_NDM (50.0%) and bla_KPC (16.7%) as the main resistance determinants. Outer membrane porin expression alterations were also common (reduction of OmpK36-associated porin bands in 62.5% of isolates and concurrent reduction of OmpK35/OmpK36-associated bands in 25.0%) and were strongly associated with higher carbapenem MICs, underscoring the multifactorial nature of resistance.

Carbapenemase production was detected in the majority of isolates (85.0% by Carba NP and 87.5% by PCR), consistent with global reports indicating that carbapenemase-mediated resistance has become the dominant mechanism among CRE worldwide [2,4]. These enzymes hydrolyze carbapenems efficiently and are commonly located on mobile genetic elements that facilitate horizontal dissemination between bacterial species [5,6]. The high prevalence of carbapenemase genes observed in the present study emphasizes the critical role of molecular surveillance in understanding local resistance epidemiology and informing infection-control strategies. The small discrepancy between phenotypic and molecular detection reflected a limited number of isolates carrying carbapenemase genes that were not detected by the Carba NP assay. Such discordance may arise because of low-level gene expression, variable enzyme activity, or reduced sensitivity of phenotypic assays in certain isolates.

The predominance of bla_NDM (60/120, 50.0%) followed by bla_KPC (20/120, 16.7%) and bla_OXA-48-like (20/120, 16.7%) is consistent with the reported epidemiology of carbapenem resistance in India. Although bla_NDM remained the predominant carbapenemase in the present study, the proportion of bla_KPC-positive isolates (16.7%) was higher than that reported in many Indian surveillance studies, where NDM and OXA-48-like enzymes typically predominate. This finding may reflect local institutional epidemiology, selective antimicrobial pressure, introduction of resistant strains through patient transfer, or unrecognized clonal dissemination within the healthcare setting. Because whole-genome sequencing and molecular typing were not performed, the underlying factors contributing to the observed bla_KPC prevalence could not be determined. These findings highlight the importance of institution-specific surveillance, as the distribution of carbapenemase genes may vary considerably between regions and healthcare facilities. Several studies from Indian healthcare settings have documented NDM as the most frequently detected carbapenemase, followed by OXA-48-like enzymes, with KPC being relatively less common [11,13]. Although bla_KPC was less frequent than bla_NDM, the observed prevalence of 16.7% indicates that KPC-producing isolates are also present in our setting and warrant continued surveillance. The coexistence of multiple carbapenemase genes was observed in 10 isolates (8.3%), most commonly bla_NDM and bla_OXA-48-like. These findings underscore the importance of institution-specific surveillance, as regional variation in carbapenemase distribution exists across India [4,10,11].

A high prevalence of ESBL genes was also observed, particularly bla_CTX-M (95/120, 79.2%), followed by bla_TEM (70/120, 58.3%) and bla_SHV (55/120, 45.8%). The frequent coexistence of ESBL and carbapenemase determinants suggests accumulation of multiple resistance mechanisms via mobile genetic elements and further complicates therapeutic options [4,6].

Porin expression alterations constituted another important resistance mechanism. Marked reduction of OmpK36, either alone (75/120, 62.5%) or in combination with OmpK35 reduction (30/120, 25.0%), was common. Isolates with both carbapenemase genes and porin expression reduction (69/120, 57.5%) exhibited significantly higher carbapenem MICs than carbapenemase-positive isolates without porin reduction (Mann-Whitney U test: for imipenem, U = 1420, Z = -5.02, p < 0.001; for meropenem, U = 1385, Z = -5.18, p < 0.001). These findings are consistent with established models in which reduced membrane permeability may contribute to higher levels of carbapenem resistance in the presence of carbapenemase enzymes. Although gene-specific stratified analyses were not performed, the observed association between porin reduction and elevated carbapenem MICs supports the contribution of combined resistance mechanisms. Future studies with larger numbers of isolates in individual carbapenemase subgroups may help clarify the relative impact of specific resistance determinants. However, because only univariate analyses were performed, the independent contribution of porin alterations relative to specific carbapenemase types could not be determined [5,6]. Notably, all carbapenemase-negative isolates (15/120, 12.5%) carried ESBL genes and showed porin expression alterations, supporting the role of non-carbapenemase mechanisms [7].

The clinical implications of these findings are substantial. Identification of specific carbapenemase classes is increasingly important for guiding therapy with newer β-lactam/β-lactamase inhibitor combinations [4,6]. Management of metallo-β-lactamase producers (such as NDM-positive isolates) remains particularly challenging [14,15]. The high proportion of CRE isolates from intensive care units (65/120, 54.2%) further highlights the need for stringent infection prevention measures [2,10,16-18].

The therapeutic relevance of carbapenemase characterization has increased with the availability of newer β-lactam/β-lactamase inhibitor combinations. KPC-producing Enterobacterales may respond to agents such as ceftazidime-avibactam and meropenem-vaborbactam, which demonstrate activity against many serine carbapenemases. In contrast, NDM-producing isolates remain more difficult to treat because metallo-β-lactamases are not inhibited by currently available β-lactamase inhibitors. Consequently, treatment strategies for NDM-producing organisms often require alternative approaches, including combination regimens or newer agents with activity against metallo-β-lactamase-producing pathogens. Given the predominance of bla_NDM in the present study, accurate molecular identification of resistance mechanisms may have important implications for antimicrobial selection and stewardship efforts.

Limitations

Several limitations warrant consideration. First, the single-center retrospective design limits generalizability. Second, carbapenemase and ESBL genes were detected by PCR without confirmatory sequencing, restricting genetic variant characterization. Third, whole-genome sequencing was not performed, precluding analysis of clonal relationships, plasmid epidemiology, and transmission dynamics. Fourth, efflux pump activity was not assessed. Fifth, porin analysis was semi-quantitative (SDS-PAGE with densitometry), and OmpK35/OmpK36 assignments were inferred primarily from expected molecular-weight characteristics and comparison with reference strains. Specific porin identities, particularly in non-*Klebsiella* isolates, were not confirmed by molecular, immunologic, or proteomic methods; therefore, findings should be interpreted as reduced major outer membrane porin expression rather than definitive identification of individual porins. Sixth, detailed PCR parameters were unavailable from archived records, limiting methodological reproducibility. Seventh, multivariable analyses and gene-specific stratified comparisons were not performed; therefore, the independent contribution of individual carbapenemase types and porin alterations to carbapenem resistance could not be fully determined. Finally, despite statistically significant differences, individual MIC values showed considerable variability. Notwithstanding these limitations, our findings indicate that carbapenem resistance in our institution is primarily associated with carbapenemase production and reduced porin expression. Continued surveillance remains essential, and future studies incorporating whole-genome sequencing are needed to understand transmission dynamics and resistance evolution [16,17].

## Conclusions

This study demonstrated that carbapenem resistance among clinical Enterobacterales isolates in our institution is strongly associated with carbapenemase production, particularly bla_NDM and bla_KPC, and is augmented by reduced outer membrane porin expression. Carbapenemase genes were detected in the majority of isolates, while porin expression reduction was associated with elevated carbapenem MICs. All carbapenemase-negative resistant isolates exhibited ESBL gene carriage and porin expression reduction, highlighting the contribution of multiple resistance mechanisms to the carbapenem-resistant phenotype.

These findings emphasize the need for routine molecular characterization alongside phenotypic susceptibility testing to accurately identify resistance mechanisms and support appropriate antimicrobial therapy. The high burden of carbapenem-resistant isolates in intensive care settings further underscores the importance of targeted infection-control measures and antimicrobial stewardship interventions. Future multicenter genomic surveillance studies are needed to define the transmission dynamics and evolutionary pathways of CRE and to guide evidence-based strategies for containment and treatment.
